# Killing Two Birds with One Stone by Administration of Soluble ACE2: A Promising Strategy to Treat Both Cardiovascular Diseases and SARS-CoV-2 Infection

**DOI:** 10.3390/v13112243

**Published:** 2021-11-08

**Authors:** Fengling Feng, Jiaoshan Chen, Jin Zhao, Yanjun Li, Minchao Li, Caijun Sun

**Affiliations:** 1 School of Public Health (Shenzhen), Shenzhen Campus of Sun Yat-sen University, Shenzhen 518107, China; fengfling@mail.sysu.edu.cn (F.F.); chenjsh59@mail2.sysu.edu.cn (J.C.); zhaoj47@mail2.sysu.edu.cn (J.Z.); liyj253@mail2.sysu.edu.cn (Y.L.); limch7@mail2.sysu.edu.cn (M.L.); 2 Key Laboratory of Tropical Disease Control, Sun Yat-sen University, Ministry of Education, Guangzhou 510080, China

**Keywords:** SARS-CoV-2, ACE2 receptor, soluble ACE2, optimized sACE2, therapeutic agent

## Abstract

The severe acute respiratory syndrome coronavirus 2 (SARS-CoV-2) enters host cells mainly by the angiotensin converting enzyme 2 (ACE2) receptor, which can recognize the spike (S) protein by its extracellular domain. Previously, recombinant soluble ACE2 (sACE2) has been clinically used as a therapeutic treatment for cardiovascular diseases. Recent data demonstrated that sACE2 can also be exploited as a decoy to effectively inhibit the cell entry of SARS-CoV-2, through blocking SARS-CoV-2 binding to membrane-anchored ACE2. In this study, we summarized the current findings on the optimized sACE2-based strategies as a therapeutic agent, including Fc fusion to prolong the half-life of sACE2, deep mutagenesis to create high-affinity decoys for SARS-CoV-2, or designing the truncated functional fragments to enhance its safety, among others. Considering that COVID-19 patients are often accompanied by manifestations of cardiovascular complications, we think that administration of sACE2 in COVID-19 patients may be a promising therapeutic strategy to simultaneously treat both cardiovascular diseases and SARS-CoV-2 infection. This review would provide insights for the development of novel therapeutic agents against the COVID-19 pandemic.

## 1. Introduction

The outbreak of coronavirus disease 2019 (COVID-19) has spread rapidly around the world, and become one of the severest public health threats in recent decades. By the end of October 2021, there have been more than 240 million confirmed cases of COVID-19, including 4.9 million deaths globally. COVID-19 was caused by severe acute respiratory syndrome coronavirus 2 (SARS-CoV-2), with clinical symptoms ranging from asymptomatic infection, mild disease, severe lung failure, multiorgan damage, and eventually to death [1]. Besides, numerous reports showed that COVID-19 patients are usually associated with manifestations such as cardiovascular complications [2,3,4]. SARS-CoV-2, along with severe acute respiratory syndrome coronavirus (SARS-CoV) and Middle East respiratory syndrome coronavirus (MERS-CoV), are members of the beta-coronavirus family. So far, compared with the SARS-CoV and MERS-CoV outbreak in 2002 and 2012, SARS-CoV-2 has caused more deaths and economic losses worldwide [5].

SARS-CoV-2 is a single-stranded positive-sense RNA virus, and shares 79% genome sequence identity with SARS-CoV and 50% genome sequence identity with MERS-CoV [6]. The entry of SARS-CoV-2 into host cells is mediated by its spike glycoprotein (S protein), which is composed of S1 and S2 functional subunits. The S1 subunit, which consists of the N-terminal domain (NTD) and the receptor binding domain (RBD), is responsible for binding to the receptor on host cells. The S2 subunit contains fusion peptide (FP), heptad repeat 1 (HR1), central helix (CH), connector domain (CD), heptad repeat 2 (HR2), transmembrane domain (TM), and cytoplasmic tail (CT), which mediates the fusion of the viral envelope with the host cell membrane [7]. The RBD of the S protein initiates the viral entry through binding to the ACE2 receptor on the surface of host cells.

ACE2 is a monocarboxylic peptidase for cleaving several peptides within the renin–angiotensin system (RAS) and other substrates, such as apelin. ACE2 is ubiquitously expressed in the heart, vessels, gut, lung (particularly in type 2 pneumocytes and macrophages), kidney, testis, and brain. Of note, the abundant surface of alveolar epithelial cells in the lung tissue might explain the vulnerability of this organ to the consequences of SARS-CoV-2 invasion. ACE2 is an important negative regulator in RAS. Besides of its well-known physiological functions in cardiovascular system, ACE2 has also been exploited as a receptor to mediate the entry into host cells of some viruses, including SARS-CoV-2, SARS-CoV, and NL63-CoV [8]. Thus, SARS-CoV-2 infection might be blocked by interfering in the interaction between the virus and ACE2.

Currently, numerous strategies are being developed to prevent and control SARS-CoV-2 infection, including the use of prophylaxis vaccines, convalescent plasma treatment, suppression of excessive inflammatory response, disruption of SARS-CoV-2 replication, and inhibition of SARS-CoV-2 fusion/entry, and so on. Nevertheless, SARS-CoV-2 is still rapidly spreading worldwide. Therefore, alternative strategies are urgently needed to control the current COVID-19 pandemic. Interestingly, different forms of recombinant human sACE2 have been reported as a potent inhibitor for SARS-CoV-2 entry [9,10,11]. This review will discuss the recent promising strategies that exploit sACE2 as bait to block SARS-CoV-2 entry, and provide some potential prospective for the development of novel therapeutic agents against SARS-CoV-2 infection.

## 2. Administration of sACE2 in COVID-19 Patients as a Promising Therapeutic Treatment for Both Cardiovascular Diseases and SARS-CoV-2 Infection

**Figure 1:**
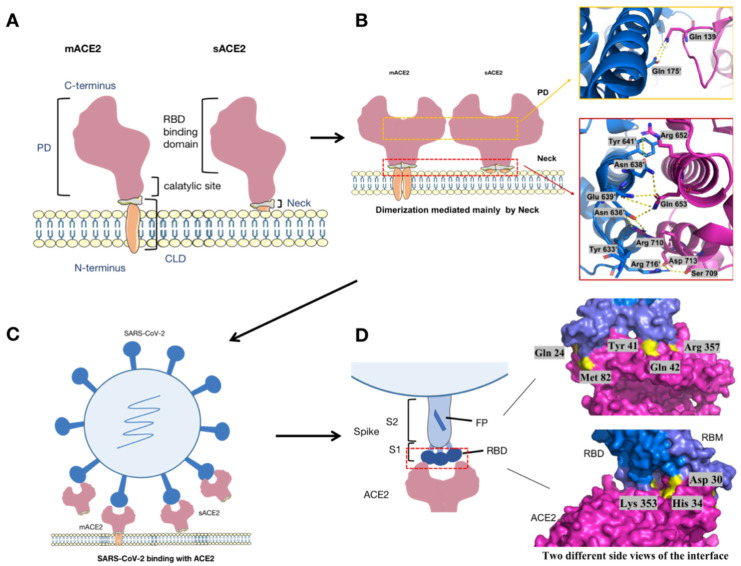
The sketch of ACE2 structure and its binding with S protein of SARS-CoV-2. (**A**) mACE2 consist of a PD domain and CLD domain, while sACE2 consist of a PD domain and the extracellular domain of CLD. (**B**) The neck and PD contribute to the homodimerization of ACE2. The blue and pink spirals represent two ACE2 dimerization protomers. (**C**) SARS-CoV-2 interacts with ACE2 through the spike protein. (**D**) The RBD domain in the S1 of SARS-CoV-2 directly interacts with the PD domain of ACE2 to form the RBD–ACE2 interface. Two different side views of the RBD–ACE2 interface are displayed on the right side of D in order to show all eight key amino acid residues on the binding interface. In the S-ACE2 binding interface map, pink represents ACE2, bright blue represents RBD of spike protein, dark blue represents the receptor binding motif RBM, and bright yellow represents the key residues labeled on the interface.

In physiological conditions, there are two forms of ACE2, full-length membrane-bound ACE2 (mACE2) and soluble ACE2 (sACE2). The former is located on the cell membrane, consisting of a transmembrane anchor and an extracellular domain, while sACE2 lacks membrane anchors and is shed into the circulation in blood [8]. Full-length ACE2 consists of lead peptide (residues 1–18), an N-terminal peptidase domain (PD, residues 19–615), and a C-terminal collectrin-like domain (CLD, residues 616–768) that ends with a single transmembrane helix and a ~40-residue intracellular segment. Between the PD and transmembrane helix is a ferredoxin-like fold domain, termed as the neck domain (residues 616 to 726), which contributes to ACE2 dimerization. The dimer formation mainly depends on polar interactions between Arg652, Arg710, Gln653, Ser709, and Asp713 in neck domain of the first ACE2 protomer and Tyr641, Tyr633, Asn638, Glu639, Asn636, and Arg716 in the neck domain of the second ACE2 protomer, with one pair of interactions between Gln139 and Gln175 on the PD dimer also contributing a weak force [12] (Figure 1A,B). Each PD accommodates one RBD when interacting with spike protein of SARS-CoV-2, and the key amino acid residue of ACE2 attributed to Tyr41, Gln42, Lys353, and Arg357 at the N terminus; Asp30 and His34 in the middle of the bridge; and Gln24 at the C terminus, with the Phe486 of the RBD interacting with the Met82 of ACE2 through van der Waals forces [12] (Figure 1C,D).

### 2.1. ACE2 Perform a Crucial Role in the Cardiovascular System through Modulating the Renin Angiotensin System

In physiological conditions and SARS-CoV infection, ADAM-17 (a disintegrin and metalloproteinase-17) can cleave mACE2 and result in sACE2 shedding, a process also associated with acute lung injury (ALI) [13]. sACE2 generated by proteolytic cleavage from the membrane anchor is normally present in plasma at a low concentration [14]. ACE2 is a potent negative regulator of the renin-angiotensin system (RAS). Thus, the RAS antagonists, such as angiotensin converting enzyme inhibitors (ACEIs) or angiotensin receptor blockers (ARBs), are commonly used to control the blood pressure of patients with hypertension through increasing the expression of ACE2 [15]. As a human homolog of ACE, ACE2 catalyzes the conversion of angiotensin II (Ang II) to angiotensin 1–7 (Ang1–7), thereby counterbalancing ACE activity [16]. Ang1–7 serve as Ang II antagonist, with the function of vasodilation, reduction of vascular permeability, anti-fibrosis, anti-inflammation, and other effects [17]. The degradation of Ang II to Ang1–7 can be blocked by selective ACE2 inhibitors like MLN-4760 [18]. Therefore, increasing ACE2 levels with medications (such as ACEIs or ARBs) may have a protective effect on COVID-19 patients through reduction in the risk of severe respiratory symptoms. In addition, plasma sACE2 levels were significantly induced in patients taking ACEIs or ARBs [19]. The increased level of extracellular sACE2 can be used as a potent decoy receptor for SARS-CoV-2 neutralization. ACE2 also converts angiotensin I (Ang I) to angiotensin 1–9 (Ang1–9), which will activate the release of bradykinin in endothelial cells, and thus have an anti-hypertrophy effect in the heart. Furthermore, Ang1–9 can also be further converted to Ang1–7 by ACE [20,21]. Studies have shown that ACE2 gene deletion in mice leads to Ang II-mediated cardiorenal fibrosis and aggravation of oxidative stress in the heart and kidney, while administration of recombinant ACE2 (rhACE2) can effectively restore cardiovascular, cardiac, and renal functions [22]. In addition, attention has been paid to the fact that the decrease in serum ACE2 activity is a selective biomarker of abnormal cardiac systolic function in the development from hypertension to heart failure [23]. The absence of ACE2 can enhance the sensitivity to heart failure, while increasing the level of ACE2 can prevent and reverse the phenotype of heart failure [24]. In fact, previous studies have shown that administration of recombinant sACE2 reduced the blood pressure by increasing the production of Ang1–7, and thus could be a promising therapeutic treatment for cardiovascular diseases [25].

Patients with hypertension, diabetic obesity, and cardiovascular disease are often accompanied with severe endothelial dysfunction, which is considered to be an important event of high-risk patients with severe COVID-19. Measuring vascular endothelial function can thus help predict severe conditions in high-risk patients with COVID-19 [26,27]. Animal studies have shown that overexpression of ACE2 contributes to endothelial protection, thus contributing to atherosclerosis [28,29]. In addition, ACE2 can be used as a regulatory factor in heart failure and can regulate cardiac function by counterbalancing the function of ACE or by Ang1–7 and Apelin. Especially, it can improve myocardial performance and cardiac remodeling through Ang1–7 [30,31,32,33].

### 2.2. SARS-CoV-2 Enters into the Host Cell via the ACE2 Receptor and Causes a Series of Deleterious Response

It is well known that SARS-CoV-2 can bind to ACE2 receptors, and subsequently initiate membrane fusion and viral entry into host cells. The first step of viral entry is the binding of the N-terminal portion of the viral protein S1 unit to a pocket of ACE2 receptor, and the S1 and S2 units of S protein must be cleaved by host cell proteases, facilitating membrane fusion and bringing the virion into the host cells. The major protease involved in SARS-CoV-2 entry are the transmembrane protease serine 2 (TMPRSS2), a member of the Hepsin/TMPRSS subfamily, and the endosomal cysteine proteases Cathepsin B and Cathepsin L (Cathepsin B/L), which are unrelated and work independently [34,35]. TMPRSS2 is reported to be expressed on the cell surface and facilitates the fusion of the virus and the cell membrane by cleaving the S protein, while Cathepsin B/L is expressed in endosome and facilitates the fusion of the virus and the endosomal membrane after the virus is endocytosed [34,36,37]. Besides, the S protein of SARS-CoV-2 also contains a furin-like cleavage site, which allows it to be cleaved by furin at the S1/S2 site and provides a gain-of-function for a more efficient spreading, enhancing SARS-CoV-2 entry into cells with relatively low expression of TMPRSS2 and/or lysosomal cathepsins [38,39]. Following binding with the ACE2 receptor, SARS-CoV-2 virus is endocytosed, and the spike proteins are primed by cellular protease. Then, the virus is released into the cytoplasm or degraded in the endocytosomes. Viruses in the cytosol generate viral genomic RNA through a replication complex and are released from cells by exocytosis [40].

Considering the influence of endolysosomes on SARS-CoV-2 infection, various stages of this endocytosis process can be targeted against SARS-CoV-2 infection. For example, chloroquine (CQ) and hydroxychloroquine (HCQ) can limit SARS-CoV-2 replication by deacidifying the endolysosomes, which would deprive the necessary acidic conditions for virus entry into host cells [41]. Tetrandrine, which is an inhibitor for the two-pole-channel (TPC) and NED-19, also effectively inhibits virus entry into host cells [42,43]. Moreover, a number of natural compounds can enhance endolysosome acidification and autophagy, and thus might enhance coronavirus degradation. These compounds include spermidine and spermine, vitamin D3, and 17-beta-estradio, among others [44,45,46,47]. It was reported that spermidine and spermine can induce autophagy through inducing 5′-AMP-activated protein kinase (AMPK) and inhibiting the mTOR signaling pathway, thus spermidine and spermine both inhibited SARS-CoV-2 infection by inducing viral degradation in endolysosomes [48]. 17-beta-estradiol is the predominant estrogen during the reproductive years; it was supposed to play a major role in antiviral therapies for SARS-CoV-2, considering the effects of 17-beta-estradiol in different systems, such as repressing the transcription virus genes, inflammation modulation, inhibition of late endosome virus trafficking, and so on.

Following the detachment of S1, the remaining S2 unit undergoes a conformational rearrangement to drive the fusion between the viral envelope and host cell membrane, with subsequent entry of the virus into cell, and release of its content [49]. The entry of SARS-CoV-2 via the ACE2 receptor often induces a series of detrimental immune responses in the host body, including complement activation, innate immune activation, inflammasome activation, pyroptosis, cytokine storm generation, and so on. This is because the binding of SARS-CoV-2 to the ACE2 receptors commonly leads to the formation of endosomes, resulting in reducing of the ACE2 expression on the cell surface, causing the RAS system to enter into the pro-inflammatory mode, inducing the body to produce more reactive oxygen species, fibrosis, collagen deposition, and producing a series of cytokines such as IL-6 and IL-8 [11]. Several studies reported that ADAM17 mediated shedding of sACE2 upon viral spike protein binding to ACE2 receptor and release of some proinflammatory cytokines such as IL-1b and TNFa owing to the activity of TNFa-converting enzyme (TACE) [50,51,52,53]. Thus, the concentrations of sACE2 may correlate with the level of systemic inflammation including metabolic syndrome, adiposity, inflammation, and liver damage correlating with elevated systemic oxidative stress-mediated endothelial dysfunction [53]. However, additional infusions of sACE2 were supposed to refill the lost sACE2 and help to balance RAS transiently, inhibit the SARS-CoV-2 infection, thus reducing the inflammation response [54].

### 2.3. sACE2 Could Be Developed as a Promising Strategy to Treat Both Cardiovascular Diseases and SARS-CoV-2 Infection

Of note, SARS-CoV-2 infection can lead to down-regulation of ACE2 level and accumulation of angiotensin II, which will trigger the inflammatory lesions in the respiratory tree (alveolar wall thickening, edema, infiltrates of inflammatory cells, and bleeding) [13,55]. Especially, downregulation of ACE2 induced by viral invasion could be more detrimental in individuals with a variable degree of ACE2 deficiency; for example, elder age, diabetes, hypertension, and prior heart diseases including heart failure [49]. Therefore, scientists have thought that the soluble form of ACE2 could be developed as a competitive interceptor of SARS-CoV-2 through blocking the interaction between S protein and the cell surface-bound-ACE2. sACE2 was reported to bind viral S protein in a similar fashion to mACE2, indicating that native sACE2 can bind S protein in extracellular space with the same affinity [56]. However, a recent study demonstrated that sACE2 contributed to SARS-CoV-2 infection and COVID-19 severity, especially in comorbid patients [54]. Man Lung Yeung and coworkers demonstrated in vitro that SARS-CoV-2 exploits receptor-mediated endocytosis to enhance cell entry through interaction between its spike with sACE2 or sACE2-vasopressin via AT1 or AVPR1B, respectively, thus adding recombinant ACE2 (rACE2) could enhance SARS-CoV-2 infectivity [57]. However, rACE2 concentrations close to the physiological range (i.e., ng/mL level) was used in these studies, insufficient to saturate endocytic recycling of the ACE2 receptor. Once sACE2 is used as a treatment strategy for COVID-19 with very high concentrations (~10–200 mg/mL of ACE2), rACE2 will exceed the concentration needed for the endocytosis recycle saturation, and the sufficient sACE2 will compete with the SARS-CoV-2–ACE2 complex to enter into host cells, thereby reducing the infectivity of SARS-CoV-2 [11,57,58].

Considering the important role of ACE2 in mediating rapid transformation of Ang II to Ang1–7, an ideal ACE2 inhibitor should act on the S-protein-binding site, but not reduce the peptidase activity of ACE2 for processing Ang II to Ang (1–7). Interestingly, a study demonstrated that high affinity binding of SARS-CoV-2 spike protein can enhance the carboxypeptidase activity and specificity of ACE2, which was clinically relevant to the pathogenesis of COVID-19 [59]. In fact, some studies have demonstrated that recombinant human sACE2 effectively inhibited the infection of SARS-CoV and SARS-CoV-2 [11,60]. The safety of recombinant human sACE2 has been verified in healthy human subjects and patients with lung disease, and its efficacy is further being evaluated in a phase 2 clinical trial in COVID-19 patients by Apeiron Biologics in European [61,62]. Besides, a clinical trial for COVID-19 treatment in Guangdong province in China (Clinicaltrials.gov#NCT04287686) proposed that mutations in ACE2 receptor interface may increase S/ACE2 interaction [63].

**Figure 2:**
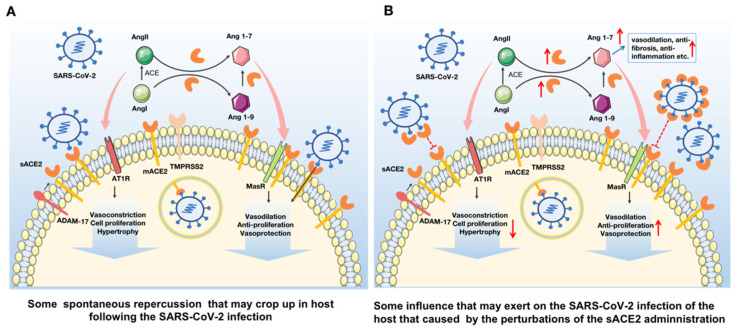
Administration of sACE2 in COVID-19 patients might play the function of killing two birds with one stone: a promising therapeutic strategy to treat both cardiovascular diseases and the SARS-CoV-2 infection. (**A**) Following the invasion of SARS-CoV-2, mACE2 will mediate its entry into host cells. Meanwhile, mACE2 and low concentrations of sACE2 in vivo will cleave Ang I and Ang II to form Ang 1-9 and Ang 1-7, respectively. Ang II promotes vasoconstriction, cell proliferation and hypertrophy by binding with AT1R while Ang1-7 promotes vasodilation, anti-cell proliferation and vasoprotection by binding with MasR. (**B**) With the administration of rsACE2, sACE2 level in vivo will increase, which will promote its binding to SARS-CoV-2 and inhibit the virus infection through blocking its further binding with mACE2. Besides, the increase of free sACE2 contributes to the further conversion of Ang I and Ang II into Ang1-9 and Ang1-7, which promotes vasodilation, anti-fibrosis and vasoprotection etc., through its effect on the RAS system. Therefore, the increase of sACE2 can not only prevent SARS-CoV-2 infection but also contribute to the treatment of cardiovascular diseases. In this picture, ADAM-17 represents a disintegrin and metalloproteinase-17; AT1R represents (Ang II)-angiotensin type 1 receptor; hACE2 represents human ACE2; TMPRSS2 represents transmembrane protease serine 2; MasR represents the Mas receptor; PD represents the N-terminal peptidase domain; and CLD represents the C-terminal collectrin-like domain. The upward red arrow represents up-regulation, and the downward represents down-regulation.

Together, administration of sACE2 in COVID-19 patients might be considered a promising therapeutic strategy to treat both cardiovascular diseases and SARS-CoV-2 infection (Figure 2).

## 3. Several Optimized Strategies to Improve the Therapeutic Effect of sACE2 against SARS-CoV-2 Infection

A strategy using full-length human sACE2-mediated inhibition against SARS-CoV-2 is currently in a phase 2 clinical trial [11]. Moreover, increasing groups have now been trying to design the optimized sACE2 fragments to enhance its affinity with S protein, or reduce its side effects using computational analysis. Herein, we summarized the current findings on different sACE2-based strategies as a therapeutic agent against SARS-CoV-2 infection (Table 1).

### 3.1. Prolonging the Half-Life of sACE2 by Fusion with a Varied Fc Fragment

Although recombinant sACE2 protein was shown to have therapeutic potential for SARS-CoV and SARS-CoV-2 infection, it exhibited a fast clearance rate with a half-life of only hours, reported by pharmacokinetic studies [55]. Therefore, it is not ideal for sustained viral inhibition in vivo [25,64]. It was reported that soluble protein fused with the immunoglobulin (Ig) constant domain Fc (fragment crystallizable) fragment can extend its residence time in plasma, gain immunoreactive functions, and improve its in vivo efficacy, so this strategy has been widely used in modern biopharmaceuticals. One study showed that recombinant protein of human sACE2, which was fused with the Fc region of the human immunoglobulin IgG1 (termed as ACE2-Ig), had high affinity to bind to the RBD of SARS-CoV and SARS-CoV-2, and thus exerted a well-desired pharmacological property. The ACE2-Ig showed potent cross-reactivity against both SARS-CoV and SARS-CoV-2 infection in vitro [63]. In addition, they also found that the inhibition effect of ACE2-Ig remained when two active-site histidine residues (residues 374 and 378) of ACE2 were modified to asparagine residues to reduce the catalytic activity [10]. However, ACE2-IgG Fc fusion protein is endowed with FcRγ binding activity, which will promote viral invasion into FcRγ-expressing phagocytic cells, leading to increased viral infection and replication, or mediate immune complex formation, causing enhanced inflammation and immunopathology, an effect termed antibody-dependent enhancement (ADE), which is detrimental to COVID-19 patients [65,66]. Many strategies have been proposed to solve this problem; for example, the catalytic site or IgG constant region of sACE2 was mutated to abrogate the FcRγ binding function. It was reported that an ACE2 mutant in catalytic site (MDR504) no longer has catalytic activity, but its ability to bind SARS-CoV-2 is significantly enhanced, and the protein is very stable in serum with a half-life of ~145 h [67]. In addition, Zhaoyong Zhang and coworkers designed an ACE2-Fc fusion protein (hACE2-Fc) that fuses the extracellular domain of hACE2 with the N-terminal of the human IgG-Fc region (IgG-Fc fragment), which showed strong broad-spectrum neutralization activity. It can not only block the entry of SARS-CoV-2, SARS-CoV, and HCoV-NL63 into host cells, but also neutralize various variants of SARS-CoV-2, including the D614G and V367F mutants, B.1.1.7 (Alpha), B.1.351 (Beta), B.1.617.1 (Kappa), and B.1.617.2 (Delta). Most importantly, hACE2-Fc has a longer half-life in vivo than soluble ACE2 [68]. Another study demonstrated that an improved soluble ACE2, termed a “microbody”, in which the ACE2 ectodomain was fused with Fc domain 3 of the immunoglobulin heavy chain, effectively inhibited the entry of SARS-CoV-2 pseudovirus and live SARS-CoV-2 in vitro and in a mouse model [69]. The microbody also inhibits the entry of β coronaviruses and virus with the variant D614G spike. This microbody is smaller than previously described ACE2-Ig Fc fusion proteins, and contains a H345A mutation in the ACE2 catalytic active site without reducing its affinity with the SARS-CoV-2 spike RBD. Furthermore, a novel recombinant sACE2, which was linked to a chimeric molecule consisting of single-domain antibodies (sdAbs) with the variable domain of camelid heavy-chain antibodies (named VHH or nanobodies), was used to potentially treat the COVID-19 disease [70]. Compared with ACE2-Fc, this sACE2-anti-CD16 VHH bi-specific molecule has several advantages, including binding to CD16 with high affinity and binding to activating receptors, with the small size of this molecule allowing rapid permeation into different tissues, which can be easily produced in large quantities in prokaryotic and eukaryotic cell lines [71,72].

### 3.2. Increasing the Affinity between sACE2 and RBD by Gene Engineering to Create High-Affinity Decoys for SARS-CoV-2

Recent data showed that the affinity of dimeric ACE2 with SARS-CoV-2 spike RBD can be dramatically improved using both computational design and screening methods. The binding affinity of the ACE2-RBD interaction depends most crucially on the side chains of six amino acid (H34, Y41, Q42, Y83, K353, and D355) on ACE2, through using the computational alanine scanning to assess the values of the predicted change in binding energy upon mutation to alanine (DDG (complex)) greater than 1 Rosetta energy unit (REU). The ACE2–RBD interface was computationally designed using a two-stage flexible protein backbone design process, and the affinity was improved up to 12-fold. Then, these designed ACE2 variants further underwent affinity maturation by random mutagenesis and selection using yeast surface display, resulting in an additional 14-fold affinity. The highest affinity of ACE2 variant contained seven amino acid changes, and was bound to RBD up to 170-fold affinity compared with wild-type ACE2. The optimized ACE2 receptor can effectively neutralize SARS-CoV-2 infection and other coronaviruses [73]. It has been reported that the polymorphism of ACE2 seriously affects the pathogenesis and spread of COVID-19, and the binding affinity between ACE2 and RBD can be effectively regulated by targeting mutations at multiple interfaces of ACE2 [74,75]. Chan and coworkers demonstrated that mutational sites existed on the interaction surface of asparagine 90-glycosylation motif that enhanced ACE2 binding to RBD, which provides a blueprint for engineering high affinity peptides to block receptor binding sites on S protein [76]. For example, hydrophobic substitutions of ACE2/T27 increased the hydrophobic packing with aromatic residues of S protein; ACE2/D30E extended an acidic side chain to reach the K417 of S protein; and aromatic substitutions of ACE2/K31 contributed to an interfacial cluster of aromatics [76]. In addition, a kind of sACE2 with three mutations (T27Y, L79T, and N330Y), named sACE22.v2.4, increased the affinity up to 35-fold, and bound to SARS-CoV-2 S (KD 600 pM) with comparable affinity to the best-known monoclonal antibodies. Moreover, sACE22.v2.4 can broadly bind to the RBDs of diverse SARS-associated beta coronaviruses that use ACE2 receptor for cell entry [76,77]. Two residues (N90 and T92) in ACE2, which together form a consensus N-glycosylation motif, are hot spots for enriched mutations. Substitutions of N90 and T92, with the exception of T92S, which maintains the N-glycan, are highly favorable to enhance the affinity for RBD binding [77].

### 3.3. Optimizing Truncated sACE2 Peptides to Enhance Its In Vivo Safety

As mentioned above, recombinant sACE2 could be a potential treatment strategy to block the SARS-CoV-2 infection. However, a recent study demonstrated that the S of SARS-CoV-2 could simultaneously interact with sACE2 and vasopressin to form a sACE2–S–vasopressin complex, which facilitated cell entry of SARS-CoV-2 via vasopressin receptor, AVPR1B [57]. Therefore, sACE2 seems to be a double-edged sword in the fight against SARS-CoV-2 infection, which can not only block viral infection, but also promote viral entry into host cells through certain ways. These findings suggested that the safety of full-length sACE2 treatment in COVID-19 patients should be carefully reconsidered.

Interestingly, the binding site to S protein is located at the interface domain in the extracellular domain of ACE2, whereas the binding site of catalytic activity for angiotensin substrates is located within a deep cleft elsewhere in the N-terminal PD of ACE2 [12,78,79]. In fact, site mutations (residues His374 and His378 to asparagine) of the ACE2 catalytic activity were found to have no effect on S protein binding to ACE2, suggesting that the binding ability of ACE2 to S protein is independent of its catalytic activity [80]. Therefore, substitutions within the angiotensin substrate-binding cleft of ACE2 are anticipated to have minimal impact on S-binding ability, but with an enhanced in vivo safety. However, it should be noted that catalytic active protein may have desirable effects for replenishing host ACE2 activity in COVID-19 patients in respiratory diseases [13].

It is proposed that the binding residues of ACE2 involved in the interaction with RBD are located at amino acid position 21–119, thus it is hypothesized that the fragment carrying all the binding residues will have better binding affinity for RBD and can better block its entry into the epithelial cells [12,79]. A truncated version of ACE2 receptor (tACE2) covering the S-binding residues has been studies of protein–protein docking and molecular dynamic simulations, and the results demonstrated that this tACE2 has an improved binding affinity for S protein, and competed to bind to SARS-CoV-2 with wild type human ACE2 receptors [81]. Moreover, E. coli would be an ideal host for its large-scale expression. Besides, owing to its low toxicity, high specificity, and ease of mass synthesis, numerous peptides have been designed as a potential therapeutic candidate against COVID-19. Given the significance of the RBD–ACE2 interaction interface for SARS-CoV-2 infection, potentially inhibitory peptides were identified to interfere with the interaction of S protein with ACE2 by bioinformatics approaches and other strategies, including rational design based on the key interacting residues with RBD–hACE2, screening peptides against the SARS-CoV-2 from available antibacterial peptide databases, and a chimeric peptide design (AC20, AC23, DBP6, and cnCoVP-1–cnCoVP-7) with high potential to block the interaction between SARS-CoV-2 RBD and hACE2, among others. In addition, the α1-helix in ACE2 is shown to be important for the SARS-CoV-2 RBD interaction, thus a 23-mer peptide derived from the α1-helix (SBP1) of ACE2 was designed. Bio-layer interferometry measurements indicated that this peptide was specifically bound to SARS-CoV-2 RBD in a high affinity, and its antiviral activity remains to be performed [82].

### 3.4. Other Strategies for sACE2-Based Therapeutic Agents against SARS-CoV-2 Infection

In addition to direct administration of recombinant sACE2 protein, other techniques to increase its accessibility will be of great help for its large-scale use in clinics. Recently, one study reported to use mRNA-based nanotherapeutics to rapidly generate circulating and mucosal decay ACE2 for the potential treatment of SARS-CoV-2 [83]. mRNA-based therapy has become so attractive because of fast synthesis, affordable production, scalable manufacturing, a transient yet high expression of protein with appropriate posttranslational modifications and folding, and so on, which have enabled the quick development of mRNA-based COVID-19 vaccines [84]. Other vectors for gene delivery, such as DNA, viral vector, nanoparticles, and so on, can also be expected for sACE2-based therapy against COVID-19 in the future.

Another alternative way to competitively inhibit SARS-CoV-2 was proposed by using small extracellular vesicles (sEVs) from engineered ACE2-overexpressing mesenchymal stromal/stem cells (MSCs) [85]. Such sEVs expressing ACE2 might not only play a decoy function against SARS-CoV-2 infection, but also prevent the downregulation of ACE2 on type II alveolar cells, and thus promote recovery in COVID-19 patients with acute respiratory distress syndrome (ARDS). In addition, a complex of sACE2 and cyclodextrin (CD), which is a type of macrocyclic molecule linked by the pyranose unit through α-1,4-glycoside chain, was designed to be capable of enclosing highly hydrophobic molecules in their hydrophobic cavities [86]. Owing to the powerful function of CD to increase water solubility and dissolution of drugs and develop a controlled drug release system, the water solubility of sACE2 was improved through the formation of the CD–sACE2 complex, which makes it meet the requirements for drug atomization inhalation. This aerosolized sACE2 would be easily inhaled directly into the lungs, thus preventing inhaled coronavirus from infecting lung cells through the respiratory tract.

In addition to blocking the binding of sACE2 with S protein, some drugs targeting ACE2 can also block viral infection. For example, human defensin 5 (HD5) reduced SARS-CoV-2 invasion by cloaking ACE2 [87]; peptide drugs SARS-BLOCK™, which mimic the SARS-CoV-2 RBD domain to bind to ACE2, showed activity against SARS-CoV-2 pseudoparticles [88]. The potential antiviral activity of the fibronectin-derived anticancer peptide ATN-161, which might bind to the RGD motif of the S protein, and to the KGD motif in ACE2, was reported to play a role in reducing SARS-CoV-2 infection [89]. In addition, the combination therapy of remdesivir and recombinant sACE2 resulted in additional effects on SARS-Co-V-2-infected cells and human stem cell-derived kidney organoids, even reducing the doses of both hrsACE2 and remdesivir to a lower and safer levels [1]. Therefore, in the future, the combination of sACE2 and other drugs may play a better blocking role. However, it remains to be investigated whether there is a synergistic effect between these drugs.

## 4. Conclusions and Perspective

An unprecedented pandemic due to SARS-CoV-2 infection is still seriously threatening our public health and social-economic development worldwide. Encouragingly, however, different forms of COVID-19 vaccines have been successfully developed within record speed, and at least 14 kinds of COVID-19 vaccines have been approved for conditional marketing or emergency use. Meanwhile, increasing neutralizing antibodies against SARS-CoV-2 have been isolated [90,91,92,93]. However, there is a rapid accumulation of escape mutations in circulating SARS-CoV-2 strains under selection pressure [61], including B.1.1.7 strain in the United Kingdom, B.1.351 in Africa, P.1 in Brazil, and recently the double mutant strain B.1.617 in India, among others, resulting in immune escape from existing neutralizing antibodies. These mutant strains may become resistant to available neutralizing antibodies, thus posing a new challenge to current antibody therapies and vaccine inoculation. In contrast, whatever it mutates, the eventual infection of these SARS-CoV-2 variants still depends on ACE2 receptor-mediated cell entry, and mutations that reduce affinity of the soluble decoy will likely also decrease affinity for the ACE2 receptor on host cells, thereby coming at the cost of diminished infectivity and virulence. Therefore, SARS-CoV-2 may have limited potential to escape sACE2-mediated inhibition. As a result, sACE2 is a potential target for broadly blocking the entry of SARS-CoV-2 variants.

In the study of SARS-CoV-2 infection, verified animal models are needed to fully reproduce the pathogenesis of the disease. Currently, promising animal infection models of SARS-CoV-2 have been reported, including a transgenic mouse model expressing human ACE2, Syrian hamster model, ferret model, cat model, and non-human primate model [68,94,95,96]. The development of these animal models is crucial for vaccine evaluation and drug screening. However, no reported animal model of SARS-CoV-2 infection fully reproduces every key feature of severe COVID-19 [97]. More reliable animal models need to be developed in the future.

Recently, many optimized strategies have been proposed to enhance the affinity between sACE2 and S protein, which has the potential to promote the sACE2-mediated inhibition effect against SARS-CoV-2 infection. However, a notable concern is that sACE2 might also mediate the cell entry of SARS-CoV-2 via forming an sACE2–S–vasopressin complex, imply that we should carefully consider the potential safety of full length sACE2 treatment in COVID-19 patients. Therefore, strategies to design the truncated functional fragments have also been developed to reduce the side effect of soluble ACE2 protein. However, as an important component of the RAS, a system composed of a wide range of peptides, enzymes, and receptors, ACE2 plays an important role in regulating the homeostatic state of the cardiovascular system. Thus, the safety of some truncated fragments and peptides often comes at the expense of their enzymatic function. Screening the shortest truncated ACE2 without losing its enzymatic activity may be an important direction for future research to be a promising therapeutic strategy to simultaneously treat both cardiovascular diseases and SARS-CoV-2 infection. As ACE2 plays many important biological roles in regulating cardiovascular function and innate immune system, it is necessary to be cautious when using ACE2 as a therapeutic target. Once the therapeutic compounds are identified and optimized, efforts should be made to develop preparations that can specifically deliver them to the target site to maximize the expected preventive or therapeutic efficacy and reduce harmful side effects. Taken together, sACE2-based therapy should be further exploited as novel therapeutic agents against SARS-CoV-2 variants.

## Figures and Tables

**Table 1:** Summary of current strategies to optimize the sACE2-based therapeutic agents against SARS-CoV-2 infection.

sACE2 Optimization Strategy	Name	Animal or Human Trials	Advantages	Disadvantages	References
Fused with varied Fc					
Fused with the Fc fragment of human immunoglobulin IgG1	ACE2-Ig	BALB/c mice exhibit desirable pharmacological properties after a single intravenous dose of the fusion proteins	Enhanced half-life; immunoreactive functions; cross-reactivity against both SARS-CoV and SARS-CoV-2.	Possibility to compromise serum stability or activate FcRγ in myeloid cells	C. Lei. et al. Nat Commun, 11 (2020).
Fused with the Fc domain 3 of immunoglobulin (Ig) heavy chain	ACE2 “microbody”	K18-hACE2 mice treated with the ACE2 microbody protein is able to prevent lethal SARS-CoV-2 disease.	Enhanced half-life; smaller than ACE2-Ig.	Possibility to cause antibody-dependent enhancement	T. Tada. et al. Cell Rep, 33 (2020).
Linked to a chimeric molecule named VHH or nanobodies	sACE2-anti-CD16 VHH	More evidence in vivo is needed.	Rapid permeation into different tissues; produced in large quantities in prokaryotic and eukaryotic cell lines.	Possibility to cause antibody-dependent enhancement	A. Sheikhi. et al. Hum Vaccin Immunother, 17 (2021).
Fused with the N terminal of human IgG-Fc region	hACE2-Fc	Both prophylactic and therapeutic hACE2-Fc treatments effectively protected Ad5-hACE2-transduced BALB/c mice from SARS-CoV-2 infection.	Enhanced half-life.	Possibility to cause antibody-dependent enhancement	Zhang, Z.et al. Cell Discov 7 (2021).
Gene engineering					
A two-stage flexible protein backbone design process	ACE2 variants	More evidence in vivo is needed	Increased affinity	More anti-viral evidence is needed; preparation is cumbersome and expensive	A. Glasgow. et al. Proc Natl Acad Sci U S A, (2020).
Deep mutagenesis	sACE22.v2.4	More evidence in vivo is needed	High affinity; cross-reactivity against diverse SARS-associated beta coronaviruses	More anti-viral evidence isneeded; preparation is cumbersome and expensive	K.K. Chan. et al. Science, 369 (2020).
Truncated ACE2 and polypeptide					
Truncated ACE2	tACE2 (21-119aa)	More evidence in vivo is needed	Enhanced binding affinity for S protein; improved safety	Antiviral activity remains to be analyzed	Basit, A. et al. J Biomol Struct Dyn, (2020).
23-mer peptide derived from α1-helix (SBP1)	ACE2(21-43aa)	More evidence in vivo is needed	Association with SARS-CoV-2 RBD with low nanomolar affinity	Antiviral activity remains to be analyzed	G. Zhang, S. et al. bioRxiv, (2020).
Screened from five antibacterial peptide databases and a chimeric peptide design approach	AC20, AC23, DBP6, and cnCoVP-1- cnCoVP-7	More evidence in vivo is needed	Ease of synthesis and modifications; low toxicity; high target specificity and selectivity	Antiviral activity remains to be analyzed	Barh, D. et al. F1000 Research 9 (2020).
